# A study protocol to investigate the management of depression and challenging behaviors associated with dementia in aged care settings

**DOI:** 10.1186/1471-2318-13-95

**Published:** 2013-09-19

**Authors:** Marita P McCabe, David Mellor, Tanya E Davison, Gery Karantzas, Kathryn von Treuer, Daniel W O’Connor

**Affiliations:** 1 School of Psychology, Deakin University, 221 Burwood Highway, Burwood, Melbourne 3125, Victoria, Australia; 2 School of Psychology and Psychiatry, Monash University, Melbourne, Australia

**Keywords:** Challenging behaviors, Depression, Aged care, Organizational barriers, Staff training

## Abstract

**Background:**

The high occurrence and under-treatment of clinical depression and behavioral and psychological symptoms of dementia (BPSD) within aged care settings is concerning, yet training programs aimed at improving the detection and management of these problems have generally been ineffective. This article presents a study protocol to evaluate a training intervention for facility managers/registered nurses working in aged care facilities that focuses on organisational processes and culture as well as knowledge, skills and self-efficacy.

**Methods:**

A Randomised Control Trial (RCT) will be implemented across 18 aged care facilities (divided into three conditions). Participants will be senior registered nurses and personal care attendants employed in the aged care facility. The first condition will receive the training program (*Staff as Change Agents – Enhancing and Sustaining Mental Health in Aged Care*), the second condition will receive the training program and clinical support, and the third condition will receive no intervention.

**Results:**

Pre-, post-, 6-month and 12-month follow-up measures of staff and residents will be used to demonstrate how upskilling clinical leaders using our transformational training approach, as well as the use of a structured screening, referral and monitoring protocol, can address the mental health needs of older people in residential care.

**Conclusions:**

The expected outcome of this study is the validation of an evidence-based training program to improve the management of depression and BPSD among older people in residential care settings by establishing routine practices related to mental health. This relatively brief but highly focussed training package will be readily rolled out to a larger number of residential care facilities at a relatively low cost.

**Trial registration:**

Australia and New Zealand Clinical Trials Register (ANZCTR): The Universal Trial Number (UTN) is U1111-1141-0109.

## Background

Mental health disorders are common in aged care settings. A review of the literature reported that almost one third of aged care residents have depressive symptoms, while an estimated 10% of aged care residents meet criteria for a current diagnosis of Major Depressive Disorder (MDD) [1]. Despite the awareness of the high prevalence of depression in the aged care population, the high occurrence and under-treatment of depression within aged care settings is concerning, with studies indicating that depressive illness is associated with increased mortality risk of chronic disease, and the requirement for higher levels of supported care [2].

In addition, more than half of the aged care population is estimated to demonstrate Behavioural and Psychological Symptoms of Dementia (BPSD), such as agitation, verbal and physical aggression, sleep disturbance, and wandering [1]. Research has demonstrated that these disturbances are not well managed by staff in the aged care sector [3]. This is of particular concern as the failure to adequately care for older people with BSPD is associated with serious adverse outcomes, including increased falls and injury, the use of restraints, social isolation, admission to a psychiatric inpatient ward, and stress and burnout among care staff [4,5].

Aged care staff have the potential to play a key role in the detection, assessment, management, and ongoing monitoring of mental health disorders among their care recipients. However, research suggests that these staff receive little training in mental health and commonly hold misconceptions about disorders such as depression and BPSD [6,7], and as a result, have demonstrated poor skills in managing residents with these disorders [8,9]. In the project described in this paper, senior clinical and organisational leaders as well as senior Registered Nurses (RNs) and Personal Care Assistants (PCAs) will participate in a transformational training program to better detect and manage depression and BPSD. The program also focuses on how to improve workplace climate and effective leadership in mental health practices. Transformational training is an approach that is specifically designed to address factors related to organisational leadership and workplace climate, and so ensure that organisational barriers to the effective implementation of training into practice are resolved [10-13]. Research has confirmed that transformational training leads to increased trust, commitment, staff engagement, perceived management support, and cohesion [11,12], which are fundamental to effective and sustained change in organisational practices [13]. This, in turn, will allow these staff to encourage, inspire and motivate other staff to internalise and routinely use strategies to manage depression and BPSD and referral practices.

The aim of this paper is to outline the hypotheses of the current study, to describe the method utilized in the study design, and the development and proposed evaluation of the *Staff as Change Agents Program* - an educational and transformational training program for professional care staff in residential aged care.

### Hypotheses and expected outcomes

It is hypothesised that the transformational training program will be superior to care as usual in terms of:

● Organisational strategies to manage depression and BPSD, and

● Staff knowledge and skills in the provision of mental health care;

● Workplace climate factors such as staff trust, teamwork/cohesion, communication and supportive workplace relations;

● Staff factors such as stress, job satisfaction, and turnover; and

● Severity of resident depression and BPSD.

It is also hypothesised that supplementing the transformational training program with clinical support from a mental health nurse specialist will lead to greater benefits than the same program without clinical support.

## Methods

### Study design

A cluster randomised controlled design will be employed in this study, with participating facilities allocated to one of three conditions. The first condition will implement the training program in six facilities. The second condition will involve the implementation of the training program in another six facilities with the additional provision of clinical support to assist staff in these facilities with the roll out of the strategies and training offered in the program. Clinical support will be provided by an experienced and appropriately trained mental health specialist who will be situated within each facility for approximately one day per week for a six month period. The third condition will include six facilities that will participate in our project as a ‘care as usual’ control group. All participating facilities will be randomly allocated to one of these three conditions, and the control condition will have access to the training program at the end of their participation in the no-intervention condition.

### Target population

A total of 108 staff will participate in the study, including 54 senior staff (i.e., 3 facility managers and/or RNs from each of the 18 facilities) and 54 senior PCAs (i.e., 3 PCAs from each of the 18 facilities).

A total of 360 residents will be recruited to participate in the study. Across each of the 18 facilities, 10 residents with dementia who present with BPSD will be selected through consultation with facility staff. Similarly, 10 residents with undetected and untreated depression will be recruited across all 18 facilities. Initially depressed residents will be identified through examination of resident files and existing documentation collected for every Australian resident (known as the Aged Care Funding Instrument [ACFI]), which includes a depression screening instrument – the Cornell Scale for Depression in Dementia [14]. However final recruitment will be based on a Research Fellow conducting the Cornell Scale and selecting residents with a Cornell score of 8 and above (must include low mood in symptom count).

### Intervention program

The four-session *Staff as Change Agents – Enhancing and Sustaining Mental Health in Aged Care* program was developed based on evidence from the researchers’ previously established training programs, previous findings in relation to organizational barriers in aged care, and current programs using transformational training in other settings [15]. An additional booster session focussed on organizational barriers to mental health care is also included four weeks after the completion of the program. The transformational training program is a multi-faceted approach that trains staff in the detection and management of depression and BPSD, and then develops leaders’ abilities to support staff through organisational change. Specifically, it trains staff in how to encourage, inspire and motivate other staff to internalise and routinely use strategies to manage depression and BPSD and referral practices. It enables staff to see these strategies as important and meaningful to residents and the teams themselves. The training also highlights the relational aspects of teams, providing leaders with training in developing trust, cohesion and communication amongst team members (see Table 1). This training approach is vastly different from the way that aged care teams typically function at present, with for example, managers often engaging in transactional behavior that try to gain staff compliance via offering rewards, or punishing deviations from procedures – a managerial style that results in emotional exhaustion, staff burnout, and poor adherence to workplace initiatives [16].

**Table 1:** **Delivery format and content of the *Staff as Change Agents – Enhancing and Sustaining Mental Health in Aged Care *training program**

**Session 1**	**Session 2**	**Session 3**	**Session 4**	**Booster session**
• Depression and Workplace Environment	• Behavior of Concerns Associated with Dementia	• Staff as Change Agents	• Sustaining Mental Health in Aged Care	• Approximately 4 weeks after session 4
• detect symptoms of depression and how to recognise masked symptoms of depression	• describe and discuss challenging behavior of residents with dementia	• explore workplace environment factors for improving mental health protocols	• identify key issues that lead to resistance to change in the detection and management of depression and BPSD	• review skills and instruments introduced in sessions one and two
• adopt effective communication and psychosocial strategies when dealing with residents with depression	• explore workplace challenges	• examine workplace outcomes, strengths and gaps		• revisit staff competencies in using these instruments
• use checklist and refer resident on to a senior staff member	• review “Assessment & Management Tool for Staff”	• define effective leadership behavior		• additional training using the Cornell Scale for Depression in Dementia and the assessment tool for staff to better manage
• administer Cornell Scale for Depression in Dementia	• discuss benefits and challenges using this assessment	• discuss key attributes and behavior for facilitating change		
define workplace environment and the factors that influence staff attitudes and behavior	explore how staff can implement this assessment tool using the enabling factors of effective communication, trust, teamwork and cohesion			challenging behavior
• identify facility’s work environment				• review mental health protocols and the sustained implementation of the instruments

### Evaluation of the training program

#### Evaluation measures for aged care staff

All of the following measures will be completed by aged care staff at the 18 residential facilities at pre-intervention, post-intervention, 6-month and 12 month follow-up (or equivalent time for the control group).

1. *Knowledge of Late Life Depression Scale – Revised*. Developed by our team with a sample of 320 care staff in our previous study, an expanded version of this questionnaire will be used to measure staff knowledge of depression and BPSD (Karantzas et al., in press). This instrument was validated with confirmatory factor analysis and has good psychometric properties. It was also sensitive to change in knowledge of depression in our pilot evaluation [4].

2. *Confidence in Working with Depressed Older People Scale.* This four-point Likert scale will be used to measure how confident staff members felt working with people with depression: ‘Not at all confident’ to ‘Very confident’.

3. *Self-Efficacy in Working with Depression Scale*. Developed in our previous study, an expanded version of this questionnaire will be used to measure staff self-efficacy in caring for residents with depression and dementia [17]. Three aspects are measured, reflecting the tasks typically required of staff in working with depressed people: ‘Detecting Depression’, ‘Responding to Depression’, and ‘Communicating with Patients’. Each subscale has strong internal reliability (Cronbach’s α > .90, and has demonstrated sensitivity to change [17].

4. *Strains in Nursing Care and Work Scale*[17]. This questionnaire contains situations and thoughts or feelings which can arise when caring for people with dementia. Staff members will rate how often they encounter these situations and feelings and, when they occur, how much stress they experience.

5. *Working Environment Scale.* This scale will be used to assess aspects of the work environment, including emotions, job satisfaction, work-related problems, views about the organisation and turnover intention.

6. *Organisational Climate Questionnaire*. This 40-item questionnaire will be used to assess eight workplace climate variables [18]. Each of these subscales has good internal consistency (α > .80).

7. *Interpersonal Communication Competence Scale-Short Form (ICCS-SF).* This 15-item measure will be used to assess workplace climate variable of communication]. This scale has been found to have excellent reliability (α > .93) (DeSanctics M, Karantzas GC: The psychometric properties of the Communication Competence Scake – short form; 2012. Unpublished manuscript).

8. *Transformational/Transactional measure.* This will be used to assess workplace behavior when working with colleagues.

9. *Trust Scale.* This scale will be used to assess current perceptions of work colleagues. This scale was adapted from the Trust Scale developed by Rempel, Holmes and Zanna [19].

10. *Readiness for Organisational Change -* This four-factor scale measures current perceptions of the organi-sation [20]. Coefficient alphas for the four factors were .94 for *Appropriateness,* .66 for *Personally Beneficial,* .87 for *Management Support* and .82 for *Change Efficacy*[20].

#### Evaluation measures for aged care residents

1. Depression referral rates to primary and specialist health services will be recorded at each data collection period. Participating staff will be asked to keep records of all such referrals. This will be validated by an examination of files on a bi-monthly basis for 12 months after the implementation of the MHCTC program.

2. The management of depression and BPSD will be assessed through a review of the residents’ files by a blinded assessor who is familiar with aged care documentation. The documentation reviewed will include the resident care plan, progress notes, medical notes and medication charts. The review will focus on records of assessment, monitoring of symptoms, medical referrals, pharmacological and non-pharmacological treatment and nurse-led strategies to manage symptoms. The review will be conducted on a bi-monthly basis for 12 months after the implementation of the MHCTC program.

3. Severity of BPSD will be assessed using the Cohen-Mansfield Agitation Inventory [21], rated by two facility staff members who typically observe the resident on the morning and afternoon shifts.

4. Severity of depression will be assessed using the Cornell Scale for Depression in Dementia [14] rated by a blinded Research Fellow.

### Procedure

Staff participants in all facilities, including the control condition, will be asked to complete survey measures prior to commencing the program and again at the conclusion of the program and at 6 and 12 month follow-up.

**Figure 1:**
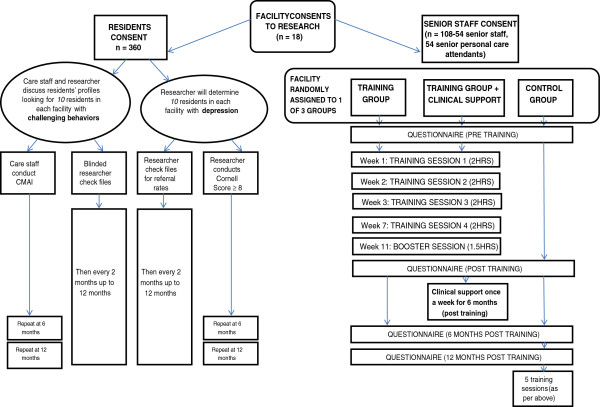
Staff as change agents – enhancing and sustaining mental health in aged care.

For the staff in the training program conditions, each training session will take approximately two hours to complete during work time, and will include discussions, role plays and handouts. The clinical support (for the six facilities in training and clinical support conditions) will assist staff in implementing the training received in the detection and management of depression and BPSD. Further, the clinician will work with staff to implement the strategies in the program to address the organizational barriers that act against the mental health needs of residents being addressed. Participants will also be asked to complete work practice activities in between sessions. The protocol adopted for the study is outlined in Figure 1.

## Results

### Data analysis

For analysis of differences in facilities at baseline, analysis of variance (ANOVA) will be used, with Fisher’s Exact test for analysing categorical variables. For changes over time, repeated measures multiple analysis of covariance (MANCOVA; controlling for group baseline scores on outcome measures) will be used for our continuous measures associated with staff outcomes. Longitudinal Configural Frequency Analysis (CFA) will be used to analyse change in categorical variables such as number of depression and BPSD referrals prior, immediate post training, at 6-month and at 12-month training follow-up. CFA is a technique analogous to repeated measures MANCOVA that allows for the estimation of trends and group differences over time but for categorical variables. Allowing for a 20% attrition rate – a rate of attrition common in our past studies – we have calculated that we will maintain at least 28 staff and 96 residents per condition at 12-month follow-up. Based on our estimated sample size in each of the conditions at 12-month follow-up, and with the expectation of a small-to-moderate effect size d = .20, the power of the analyses for aged care resident data is calculated at 0.99, and for the staff data at .91 at the two-sided significance level of α = .05 for both our mean difference testing and categorical analyses [22].

### Consent and ethics

All participating aged care staff and aged care residents will be provided with a plain language statement and will be asked to give written informed consent to participate. For staff this will be to participate in the staff training and questionnaire completion. Informed consent to examine resident documentation at the facility and collect outcome data will be obtained from the residents themselves, or if they are unable to provide consent, from their family or guardian. Staff will be paid for both the time spent in the training program as well as for the completion of the questionnaires. Upon completion of the study, participating aged care facilities will receive a plaque and staff members will receive individual certificates. The project has been approved by the Deakin University Human Research Ethics Committee (HEAG- H120_2012 and DU-HREC 2013–013).

## **Discussion**

Organisational barriers clearly limit the effective translation of skills into the routine care practices of staff to address depression and BPSD among residents in aged care facilities. The project described in this article will implement and evaluate two interventions that are designed to address the organisational barriers to mental health care (transformational staff training program and clinical support). It is expected that the project will demonstrate that with the transformational training program, a more accurate identification of depression and the causes of BPSD will ensue, resulting in appropriate treatment being delivered. This will result in less distress and improved quality of life for both residents and staff, and improve the job satisfaction of aged care staff, thereby reducing staff turnover. It will also determine the extent to which external specialist clinical support is required to produce optimal outcomes, an issue that to date has not been clearly determined, and which is essential for appropriate service planning.

## Conclusions

The model for best practice that is identified through this study will provide a framework for the development of training programs for staff to address mental health issues among residents in organisations in a sustainable way. The significance of this project goes beyond the level of individuals and facilities to the broader community. The outcomes from this study will be able to be used strategically to inform policy and practice for improved quality of care of older people with depression and BPSD more generally.

## Competing interests

The authors declare that they have no competing interests.

## Authors’ contributions

All authors have contributed to the implementation and design of this study. All authors read and approved the final manuscript.

## Pre-publication history

The pre-publication history for this paper can be accessed here:

http://www.biomedcentral.com/1471-2318/13/95/prepub
